# Retracted: Antiaging Properties of a Grape-Derived Antioxidant Are Regulated by Mitochondrial Balance of Fusion and Fission Leading to Mitophagy Triggered by a Signaling Network of Sirt1-Sirt3-Foxo3-PINK1-PARKIN

**DOI:** 10.1155/2022/9793504

**Published:** 2022-08-13

**Authors:** Oxidative Medicine and Cellular Longevity

*Oxidative Medicine and Cellular Longevity* has retracted the article titled “Antiaging Properties of a Grape-Derived Antioxidant Are Regulated by Mitochondrial Balance of Fusion and Fission Leading to Mitophagy Triggered by a Signaling Network of Sirt1-Sirt3-Foxo3-PINK1-PARKIN” [1] due to errors identified with the figures.

The concerns are related to Figures 7 and 2:

- There are 4 lanes present in Figure 7a Foxo3a where there should be 3
- Inconsistencies in Figure 2 relating to the alignment of merged images and unclear changes between submitted and published versions of the figure

When asked for a response and the original images, the authors responded that they were unable to provide the original images but were able to provide an earlier version of the manuscript where Figure 7 Foxo3a has 3 lanes. They could not explain why the image had changed during review.

Upon assessment of the published article and the files provided by the authors, the editorial board has concluded that the retraction of the article is required due to concerns regarding the validity of the images and therefore, of the results and conclusions. The authors do not agree to the retraction.
